# Supplementary material for: Characterization of Lipopolysaccharide Effects on LRRK2 Signaling in RAW Macrophages
Source: Int J Mol Sci. 2023 Jan 13;24(2):1644. doi: 10.3390/ijms24021644 (PMC9865464; doi:10.3390/ijms24021644)
Supplement: Supplementary file 1 [file ijms-24-01644-s001.zip › ijms-2007608-supplementary.pdf]

Supplementary Data

SUPPLEMENTARY TABLE

Table S1: Mouse mRNA primer sequences

Primer	Sequence
**IL-1β- Forward**	5'- TGC CAC CTT TTG ACA GTG A -3'
**IL-1β- Reversed**	5'- ATG TGC TGC TGC GAG ATT T -3'
**IL-6 - Forward**	5'- GGG ACT GAT GCT GGT GAC AT -3'
**IL-6 - Reversed**	5'- TGC CAT TGC ACA ACT CTT TTC TC -3'
**COX2 - Forward**	5'- TGA GTA CCG CAA ACG CTT C -3'
**COX2 - Reversed**	5'- CAG CCA TTT CCT TCT CTC C -3'
**NOS2 - Forward**	5'- GGG ACT GAG CTG TTA GAG ACA C -3'
**NOS2 - Reversed**	5'- TCT TGT ATT GTT GGG CTG AGA ACA -3'
**TNFα - Forward**	5'- TAC TGA ACT TCG GGG TGA TTG GTC C -3'
**TNFα - Reversed**	5'- CAG CCT TGT CCC TTG AAG AGA ACC -3'
**β-actin - Forward**	5'- ATC GTG CGT GAC ATC AAA GA -3'
**β-actin - Reversed**	5'- ATG CCA CAG GAT TCC ATA CC -3'
**RPL13A - Forward**	5'- AGA AGC AGA TCT TGA GGT TAC GG -3'
**RPL13A – Reversed**	5'- GTT CAC ACC AGG AGT CCG TT -3'

SUPPLEMENTARY FIGURES

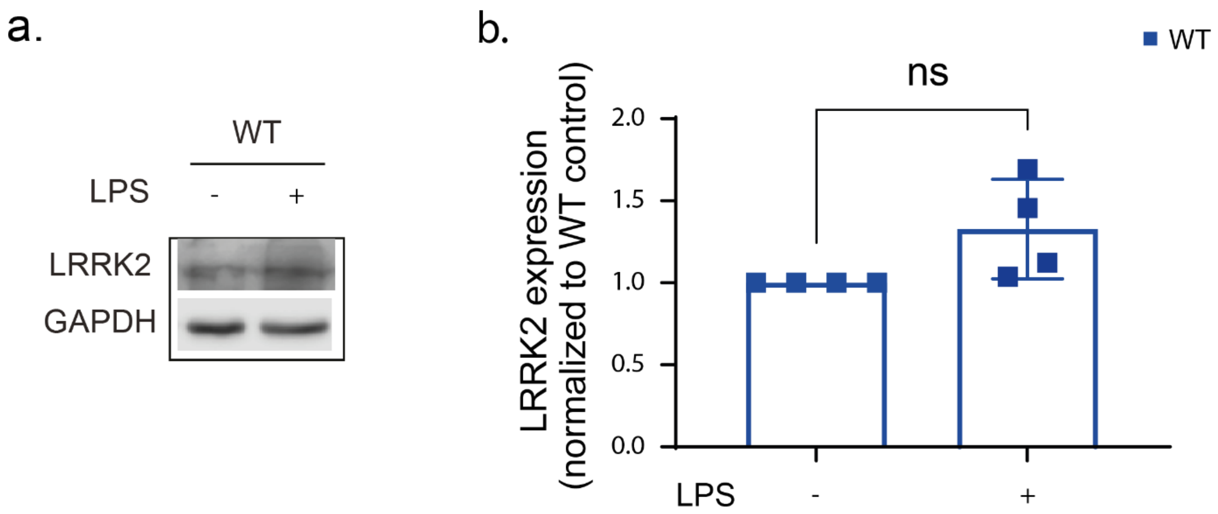

Figure S1: LRRK2 expression is not significantly affected by LPS stimulation in the WT cells. a. Representative western blot for lysates from WT cells with and without LPS stimulation for 24 hours immunoblotted with LRRK2 antibody and GAPDH antibody as a loading control. **b.** Fold change in LRRK2 expression in the WT cells stimulated with LPS for 24 hours and normalized to the WT control The experiments were performed 4 times and t-test was used to compare LRRK2 expression. Error bar represents mean ± SD. p-values indicating statistically significant differences between the mean values are defined as follows: ns not significant, $^{*}p < 0.05$, $^{**}p < 0.01$, $^{***}p < 0.001$ and $^{****}p < 0.0001$.

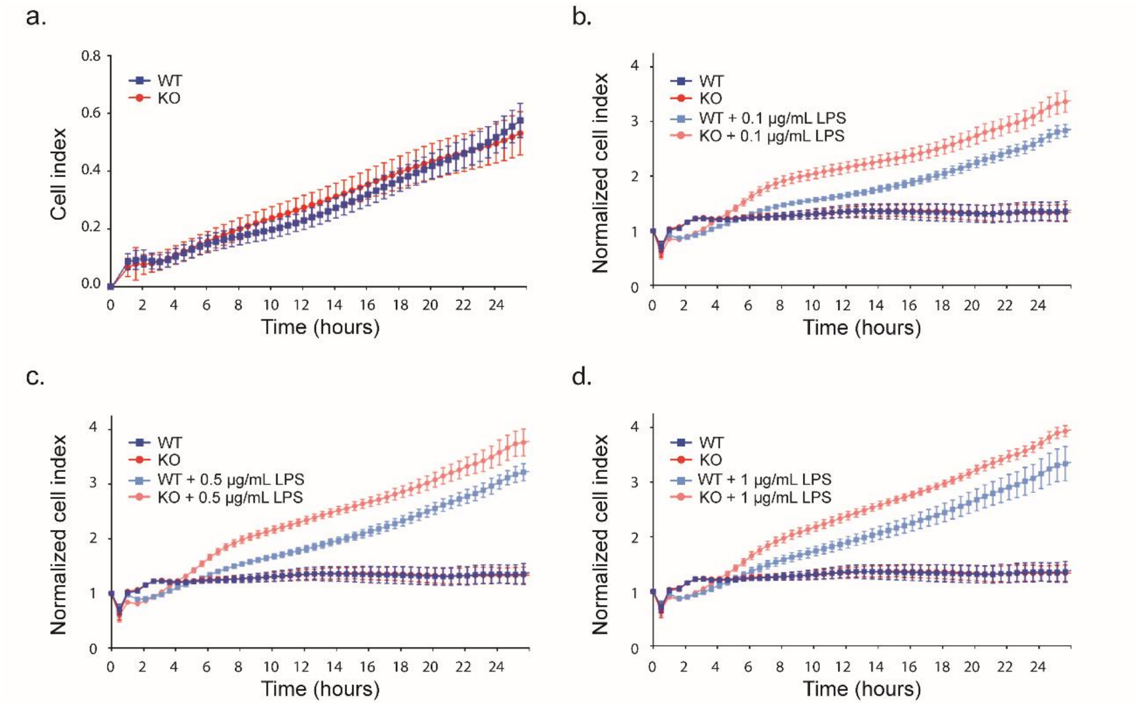

Figure S2: LPS increases the size of both the WT and KO cells yet more in the KO cells. Real-time impedance measurement in the WT and KO cells using xCELLigence system. **a.** Representative measurement of the WT and KO cells in the first 24 hours after seeding the cells showing no difference between the WT and KO cells in the morphology under basal conditions. **b-d.** xCELLigence measurement in the WT and KO cells with increasing concentration of LPS. b. 100 ng/mL LPS, c. 500 ng/mL LPS and d. 1µg/mL LPS.

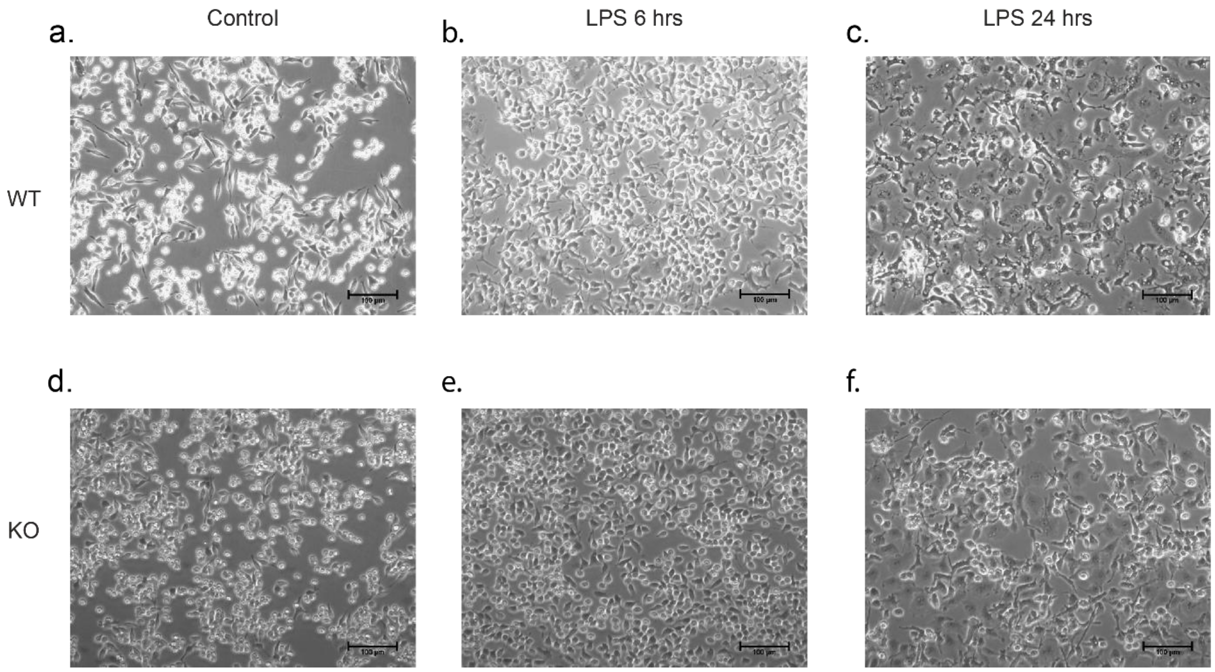

Figure S3: LPS increases the size of both the WT and KO cells without affecting the cell division. Bright-field microscopy representative pictures showing WT (a-c) and KO (d-e) cells without LPS stimulation (a & d) and after 6 (b & e) and 24 (c & f) hours for 250 ng/mL LPS. Magnification 10X. Scale bar for all images is 100 µm.

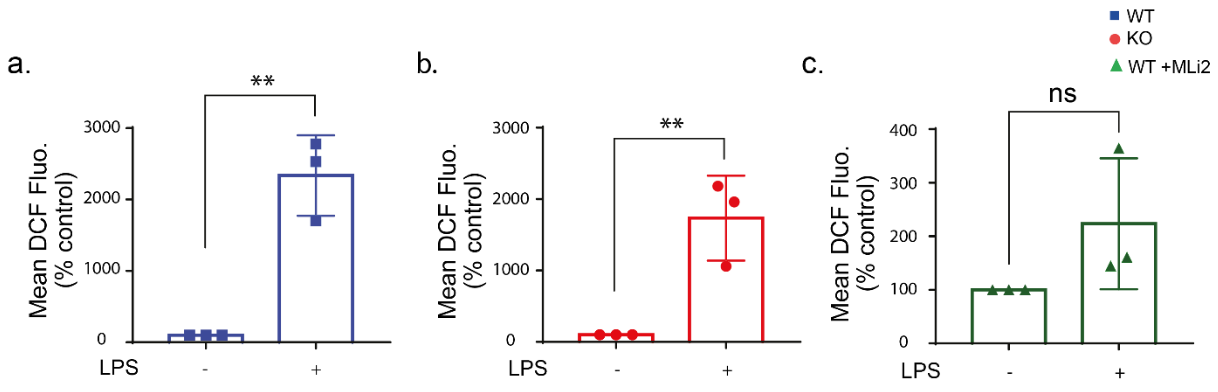

Figure S4: ROS levels are increased in WT and KO cells upon LPS stimulation. Mean DCF fluorescence in **a.** WT, **b.** KO and **c.** kinase inhibited WT cells after 24 hours of LPS stimulation. The experiment was performed 3 times and t-test was used to compare the average of the mean DCF in the cells after LPS stimulation compared to the unstimulated cells. Error bar represents mean ± SD. p-values indicating statistically significant differences between the mean values are defined as follows: ns not significant, $^{*}p < 0.05$, $^{**}p < 0.01$, $^{***}p < 0.001$ and $^{****}p < 0.0001$.

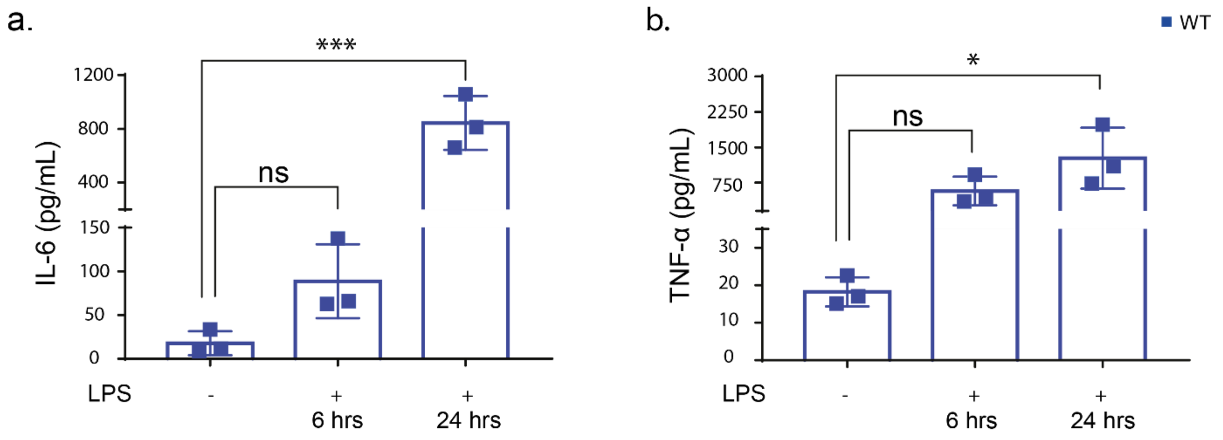

Figure S5: IL-6 and TNF-α levels in the WT cells upon LPS stimulation. a. IL-6 levels are significantly increased in the WT cells after 24 hours of LPS stimulation. **b.** TNF-α levels are significantly increased in the WT cells after 24 hours of LPS stimulation. The experiment was performed 3 times and one-way ANOVA was used to compare the average amount of IL-6 and TNF-α in the WT after LPS stimulation. Error bar represents mean ± SD. p-values indicating statistically significant differences between the mean values are defined as follows: ns not significant, $^{*}p < 0.05$, $^{**}p < 0.01$, $^{***}p < 0.001$ and $^{****}p < 0.0001$.

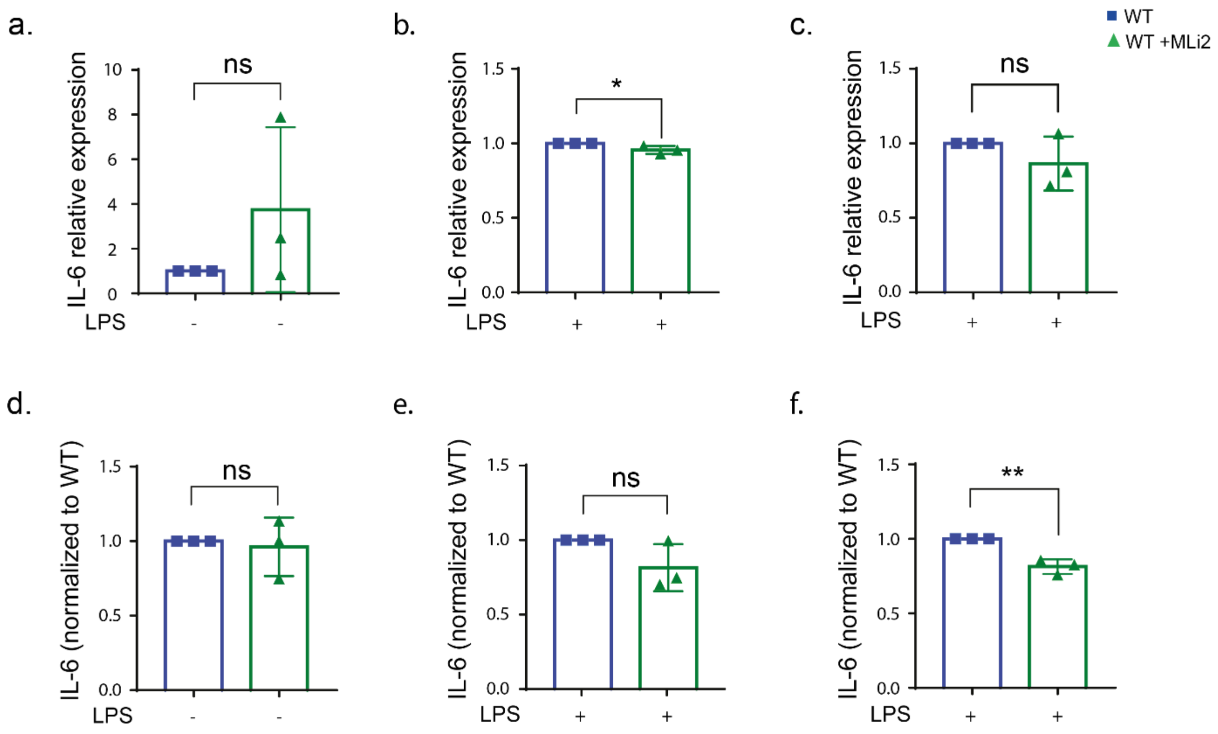

Figure S6: IL-6 expression is reduced upon the inhibition of LRRK2 kinase activity after LPS stimulation. a - c. IL-6 expression in the WT and kinase-inhibited WT cells under control conditions **(a)** and after LPS stimulation for 6 **(b)** and 24 hours **(c)** using reverse transcription quantitative real-time PCR using β-actin and RPL13A as housekeeping genes. **d - f.** The amount of IL-6 in the supernatant of the WT and kinase-inhibited WT cells under control conditions **(d)** and after LPS stimulation for 6 **(e)** and 24 hours **(f)** using ELISA assay. The experiment was performed 3 times and t-test was used to compare the average IL-6 expression or level relative to the corresponding WT. Error bar represents mean ± SD. p-values indicating statistically significant differences between the mean values are defined as follows: ns not significant, $^{*}p < 0.05$, $^{**}p < 0.01$, $^{***}p < 0.001$ and $^{****}p < 0.0001$.

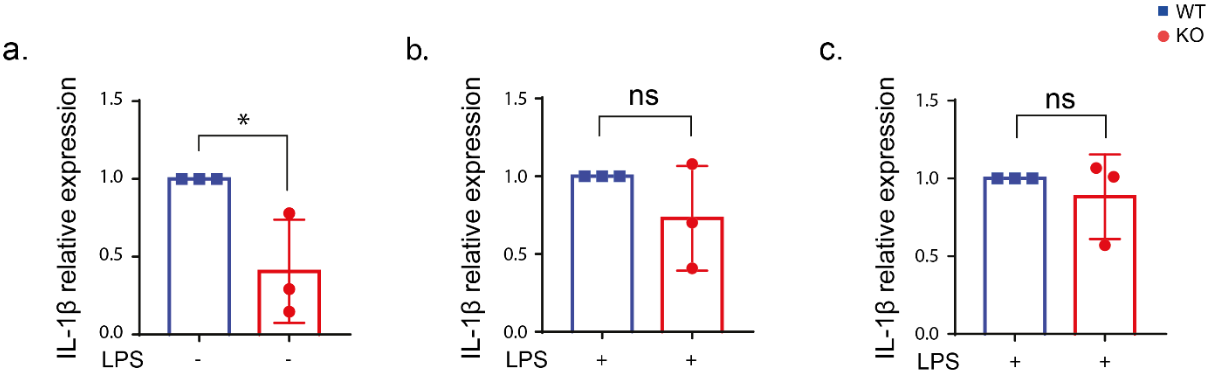

Figure S7: IL-1β expression is not changes upon LPS stimulation. Reverse transcription quantitative real-time PCR of IL-1β expression in the WT and KO cells under control conditions **(a)** and after LPS stimulation for 6 **(b)** and 24 hours **(c)**. β-actin and RPL13A were used as housekeeping genes. The experiment was performed 3 times and t-test was used to compare the average IL-1β expression relative to the corresponding WT. Error bar represents mean ± SD. p-values indicating statistically significant differences between the mean values are defined as follows: ns not significant, $^{*}p < 0.05$, $^{**}p < 0.01$, $^{***}p < 0.001$ and $^{****}p < 0.0001$.
